# Combination of extracorporeal membrane oxygenation and continuous renal replacement therapy in critically ill patients: a systematic review

**DOI:** 10.1186/s13054-014-0675-x

**Published:** 2014-12-08

**Authors:** Han Chen, Rong-Guo Yu, Ning-Ning Yin, Jian-Xin Zhou

**Affiliations:** Department of Critical Care Medicine, Beijing Tiantan Hospital, Capital Medical University, Beijing, 100050 China; Surgical Intensive Care Unit, Fujian Provincial Clinical College of Fujian Medical University, Fuzhou, 350001 Fujian China

## Abstract

**Introduction:**

Extracorporeal membrane oxygenation (ECMO) is used in critically ill patients presenting acute cardiac and/or pulmonary dysfunctions, who are at high risk of developing acute kidney injury and fluid overload. Continuous renal replacement therapy (CRRT) is commonly used in intensive care units (ICU) to provide renal replacement and fluid management. We conducted a review to assess the feasibility, efficacy and safety of the combination of ECMO and CRRT and to illustrate the indications and methodology of providing renal replacement therapy during the ECMO procedure.

**Method:**

We searched for all published reports of a randomized controlled trial (RCT), quasi-RCT, or other comparative study design, conducted in patients undergoing ECMO plus CRRT. Two reviewers independently selected potential studies and extracted data. We used the modified Jadad scale and the Newcastle-Ottawa for quality assessment of RCTs and non-RCTs, respectively. Statistical analyses were performed using RevMan 5.2.

**Results:**

We identified 19 studies meeting the eligibility criteria (seven cohort, six case control, one historically controlled trial and five studies of technical aspects). There are three major methods for performing CRRT during ECMO: ‘independent CRRT access’, ‘introduction of a hemofiltration filter into the ECMO circuit (in-line hemofilter)’ and ‘introduction of a CRRT device into the ECMO circuit’. We conducted a review with limited data synthesis rather than a formal meta-analysis because there could be greater heterogeneity in a systematic review of non-randomized studies than that of randomized trials. For ECMO survivors receiving CRRT, overall fluid balance was less than that in non-CRRT survivors. There was a higher mortality and a longer ECMO duration when CRRT was added, which may reflect a relatively higher severity of illness in patients who received ECMO plus CRRT.

**Conclusions:**

The combination of ECMO and CRRT in a variety of methods appears to be a safe and effective technique that improves fluid balance and electrolyte disturbances. Prospective studies would be beneficial in determining the potential of this technique to improve the outcome in critically ill patients.

**Electronic supplementary material:**

The online version of this article (doi:10.1186/s13054-014-0675-x) contains supplementary material, which is available to authorized users.

## Introduction

Extracorporeal membrane oxygenation (ECMO) is a lifesaving technique used in critically ill patients presenting acute cardiac and/or pulmonary dysfunctions, who are at high risk of developing acute kidney injury (AKI) and fluid overload (FO). The AKI severity can be stratified using the RIFLE [1,2] (risk, injury, failure, loss, and end-stage) classification definitions, which include changes in urine output and serum creatinine. Previous studies using the RIFLE definition in ECMO patients demonstrated that the incidence of AKI exceeded 70% [3-6], and these studies also suggest an association between poor outcomes and AKI.

Renal replacement therapy (RRT) consists of a broad range of techniques. A distinction can be made based on membrane permeability, method of molecular clearance (diffusion or convection or a combination of both) and the duration of treatment and equipment used [7]. The nomenclature of an entire continuous renal replacement therapy (CRRT) mode is based on the type of vascular access and the primary method of molecular clearance. For example, CAVH represents continuous arteriovenous hemofiltration. Although CRRT was initially developed using arterial and venous access, the pump-driven venovenous access is widely used and has now replaced the use of arteriovenous access. Peritoneal dialysis (PD) is another method that can be included in CRRT, but it is very seldom used for the treatment of AKI in patients admitted to intensive care units (ICU) [7].

CRRT is commonly used in ICUs to provide an easily initiated and efficient method of renal replacement and fluid management. The combination of ECMO and CRRT seems to be a good method for treating ECMO patients who have developed AKI. However, previous studies show wide variations in practice regarding RRT during ECMO [8]. The combination of ECMO and CRRT has been reviewed [9], but to date, no review has been conducted. Therefore, we presented this review to illustrate the methodology of providing RRT during the ECMO procedure, and to assess the feasibility, efficacy and safety of the combination of these two types of therapy.

## Methods

### Study identification

We conducted a systematic review of published reports of a randomized controlled trial (RCT), quasi-RCT, or other comparative study design, conducted in patients undergoing ECMO plus CRRT (including continuous venovenous hemofiltration (CVVH), continuous venovenous hemodialysis (CVVHD), continuous venovenous hemodiafiltration (CVVHDF), continuous arteriovenous hemofiltration (CAVH), continuous arteriovenous hemodialysis (CAVHD), continuous arteriovenous hemodiafiltration (CAVHDF) and slow continuous ultrafiltration (SCUF). Studies that introduced the technical aspects of the combination of ECMO and CRRT were also included. The exclusion criteria were: 1) studies reported the application of CRRT prior to ECMO; 2) studies reported the application of ECMO plus intermittent RRT; 3) animal experiments; and 4) case report or case series.

**Figure 1:**
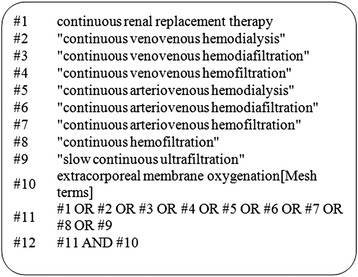
**PubMed search strategy.**

We searched the major international medical bibliographical databases: Medline (via PubMed), Web of Science, Cumulative Index of Nursing and Allied Health Literature (CINAHL), ProQuest Health & Medical Complete and the OvidSP database which includes the ACP Journal Club, Cochrane Central Register of Controlled Trials, Cochrane Database of Systematic Reviews, Cochrane Methodology Register, Abstracts of Reviews of Effects, Health Technology Assessment, NHS Economic Evaluation Database and BIOSIS Previews. We also searched trial registries for ongoing trials. We used text words or Medical Subject Headings (MeSH) headings containing ‘continuous renal replacement therapy’, ‘continuous venovenous hemodialysis’, ‘continuous venovenous hemodiafiltration’, ‘continuous venovenous hemofiltration’, ‘continuous arteriovenous hemodialysis’, ‘continuous arteriovenous hemodiafiltration’, ‘continuous arteriovenous hemofiltration’, ‘continuous hemofiltration’, ‘slow continuous ultrafiltration’ and ‘extracorporeal membrane oxygenation’ in the search. The PubMed search strategy is presented as an example in Figure 1. We also searched personal files and reference lists. The search was performed independently by two investigators (HC, RGY) and was completed on 20 December 2013, with no restriction of publication status, date or language.

### Study selection and quality assessment

Two reviewers (HC, RGY) independently screened retrieved database files and the full text of potentially eligible studies for relevance. Foreign language papers were translated if necessary. Disagreements were resolved by consensus or by discussion with another investigator (JXZ). We used the modified Jadad scale [10] for quality assessment of RCTs, and the Newcastle - Ottawa quality assessment scale (NOS) [11] for quality assessment of non-RCTs. The NOS was developed for cohort and case control studies. The NOS is categorized into three dimensions: selection, comparability and outcome (cohort studies) or exposure (case control studies). A rating between zero and nine stars is used for a semi-quantitative assessment of studies, where five or more indicates a high quality.

### Data extraction

The following data were extracted for each trial: author and year of publication, study type, study population and number, technical parameters, indicators of ECMO, indicators of CRRT, main outcome and complications. Technical parameters included the method of combining ECMO and CRRT, anticoagulation strategy, ECMO canal position, ECMO pump type and blood flow rate, ECMO modality selection (venovenous ECMO (VV-ECMO) or venoarterial ECMO (VA-ECMO)) and CRRT mode selection. The outcomes assessed included mortality, ECMO duration, fluid balance, complications and renal function recovery.

### Data synthesis

Statistical analyses were performed using RevMan 5.2. Odds risks ((OR) with 95% confidence intervals (CI)) were calculated for dichotomous data. Outcome measures were quantitatively summarized, if possible, using a random effects model. Heterogeneity among combined study results was assessed by the degree of inconsistency (*I*^2^) [12]. A value of 0% indicates no observed heterogeneity, and increasing values of *I*^2^ reflect increasing heterogeneity; a value of >25% shows at least low-to-moderate heterogeneity. When the degree of statistical heterogeneity was greater than this threshold, we investigated possible explanations by using sensitivity analysis.

## Results

**Figure 2:**
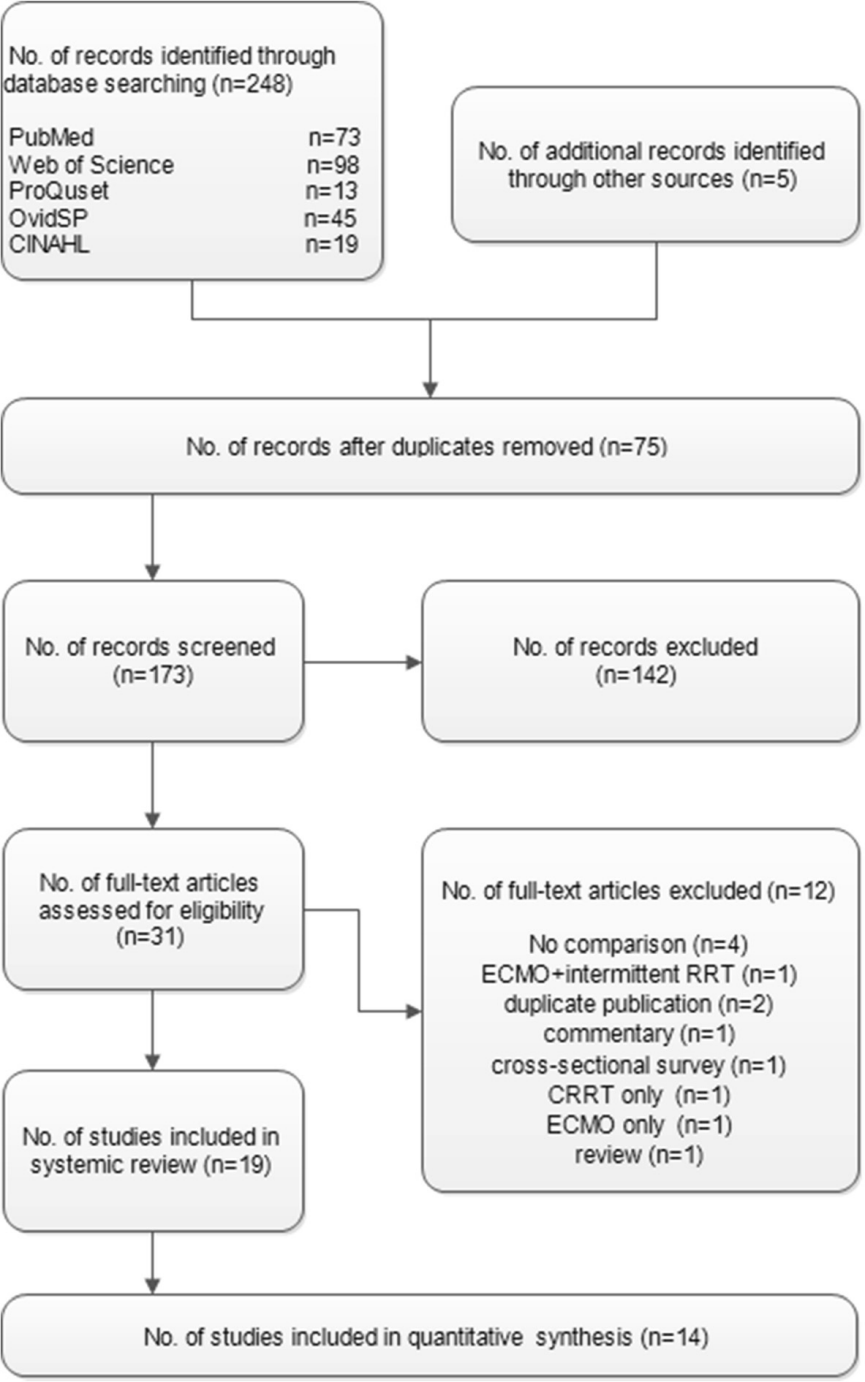
**Process for identification of the included studies.**

Figure 2 shows the results of the search and selection processes. There were 173 citations after de-duplication with EndNote X5, of which we excluded 142 citations after examining the title and abstract. Thirty-one citations were obtained in full text and 12 of these were excluded: four investigated patients who underwent ECMO plus CRRT, but without comparison, one with ECMO plus intermittent RRT, two with duplicate publication, one with commentary, one cross-sectional survey, one with CRRT only, one with ECMO only and one review. In total, 19 studies were included, consisting of seven cohort studies [13-19], six case control studies [20-25], one historically controlled trial [26] and five studies of technical aspects [27-31]. Seventeen studies were published in English [13-23,26] and two in Chinese with English abstracts [24,25]. Since no RCTs were found, quality assessment was performed using the NOS, while studies of technical aspects were not assessed. Five studies were assigned five stars [20-22,24,25], one study was assigned six stars [23], six studies were assigned seven stars [13-15,17-19] and two studies were assigned nine stars [16,26]. These studies are summarized in Table 1. Additional file 1 shows the technical parameters of ECMO and CRRT in these studies.

**Table 1:** **Study features and quality assessment**

**Study feature**						**NOS score**									
**Author, year, ref.**	**Design**	**Age of patients**	**Included period**	**Included disease**	**Number of patients**	**S1**	**S2**	**S3**	**S4**	**C1**	**C2**	**E1/O1**	**E2/O2**	**E3/O3**	**Total**
Goto, 2011 [20]	Case control study	No restriction	Apr 2002 to Feb 2011	Heart disease	14	1	0	1	1	0	0	1	1	0	5
Hamrick, 2003 [21]	Case control study	Children, one year	1990 to 2001	Congenital heart disease	53	1	0	1	1	0	0	1	1	0	5
Kolovos, 2003 [23]	Case control study	Children	Jul 1995 to Jun 2001	Congenital heart disease after surgery	74	1	1	1	1	0	0	1	1	0	6
Luo, 2009 [24]	Case control study	Adult	Feb 2005 to Jun 2008	Heart disease after surgery, chronic heart failure	45	1	0	1	1	0	0	1	1	0	5
Luo, 2010 [25]	Case control study	Adult	Jul 2005 to Jul 2009	CPR patients	11	1	0	1	1	0	0	1	1	0	5
Yap, 2003 [22]	Case control study	Adult	Dec 1998 to Jun 2001	Heart disease	10	1	0	1	1	0	0	1	1	0	5
Betrus, 2007 [13]	Retrospective cohort study	Children	1993 to 2001	Congenital heart disease after surgery	42	1	1	1	1	0	0	1	1	1	7
Cavagnaro, 2007 [14]	Retrospective cohort study	Children, one year	May 2003 to May 2005	No restriction	12	1	1	1	1	0	0	1	1	1	7
Gbadegesin, 2009 [15]	Retrospective cohort study	Children, ≤3 years	Jan 2000 to Apr 2005	Congenital heart disease	104	1	1	1	1	0	0	1	1	1	7
Hoover, 2008 [16]	Retrospective cohort study	Children ≥1 month 18 years	1990 to 2006	Respiratory failure	52	1	1	1	1	1	1	1	1	1	9
Paden, 2011 [17]	Retrospective cohort study	Children, ˂18 years	May 1997 to May 2007	Respiratory failure, heart failure	378	1	1	1	1	0	0	1	1	1	7
Ricci, 2012 [18]	Prospective cohort study	Children	NR	Heart disease	10	1	1	1	1	0	0	1	1	1	7
Wolf, 2013 [19]	Retrospective cohort study	Children	Jan 2002 to Dec 2011	Heart disease	153	1	1	1	1	0	0	1	1	1	7
Blijdorp, 2009 [26]	Historically controlled trial	Neonatal	Oct 2002 to Oct 2006	No restriction	61	1	1	1	1	1	1	1	1	1	9

C, comparability; CPR, cardiopulmonary resuscitation; E, exposure; NOS, Newcastle-Ottawa quality assessment scale; NR, not reported; O, outcome; ref., reference; S, selection.

### Systematic review

#### Technical aspects of combining ECMO and CRRT

**Figure 3:**
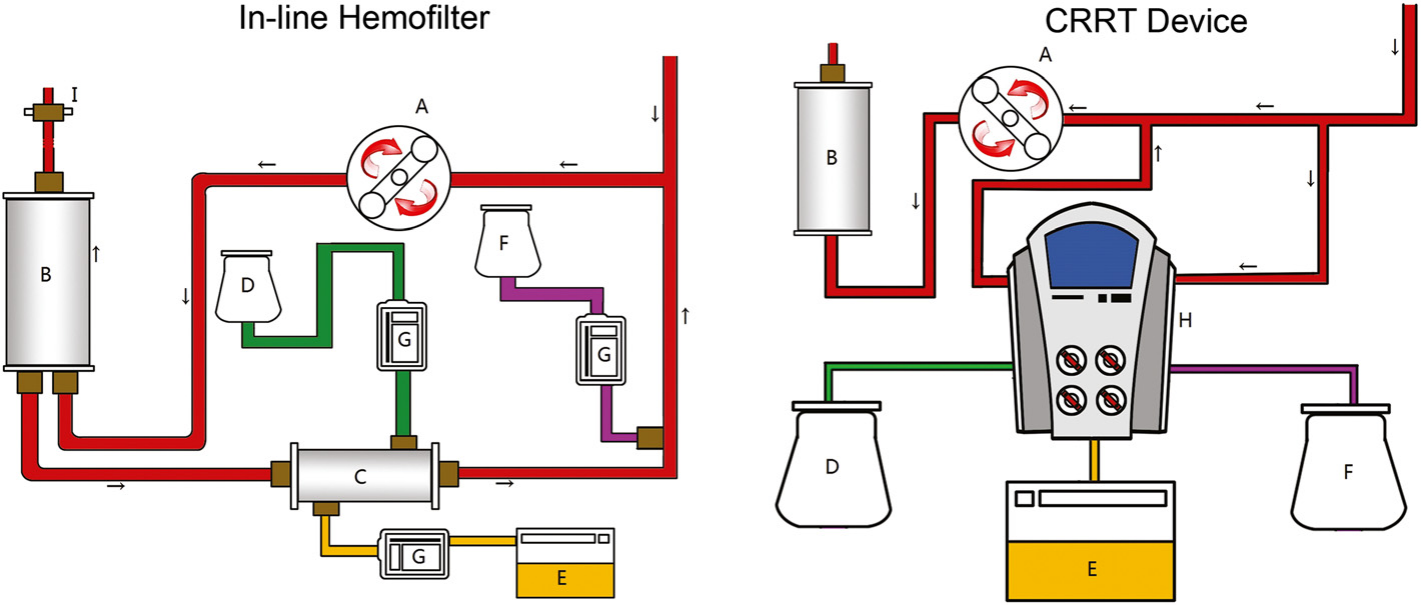
**Two typical methods of combining ECMO and the CRRT circuit.** A = ECMO pump; B = Oxygenator; C = Hemofilter; D = Dialysis fluid; E = Effluent fluid; F = Replacement fluid; G = Intravenous pump; H = CRRT device; I = Ultrasonic flow probe. CRRT, continuous renal replacement therapy; ECMO, extracorporeal membrane oxygenation.

There are a number of methods for performing CRRT during ECMO, which can be classified in three major ways: performing RRT through venous access independent of the ECMO circuit, introduction of a hemofiltration filter into the ECMO circuit using intravenous infusion pumps to control the ultrafiltrate volume and inclusion of a CRRT device in the ECMO circuit (Figure 3).

##### *Independent CRRT access*

The simplest way to perform CRRT is through venous access independent of the ECMO circuit. However, if the patient has not undergone previous cannulation of a central venous line, the situation is complicated because anticoagulation increases the risks of bleeding. Poor catheter drainage may also occur during the CRRT period.

##### *Introduction of a hemofiltration filter into the ECMO circuit (in-line hemofilter)*

The introduction of a hemofiltration filter into the ECMO circuit is the most widely used method of CRRT and has the advantage of being relatively simple and inexpensive. The hemofilter is placed after the pump to provide forward blood flow. The filter inlet is connected after the pump and the outlet is reconnected to the ECMO circuit to allow return of the blood flow to the proximal limb of the ECMO circuit. Because of the existence of a shunt, there could be a gap between the measured flow and the flow being delivered to the patient (which indicates the hemofilter blood flow rate). An ultrasonic flow probe should be placed on the arterial line of the ECMO circuit to determine the actual flow delivered to the patient.

The amount of replacement fluid, dialysis and effluent fluid is controlled by the intravenous infusion pumps included in the circuit. All methods of molecular clearance are not meant to exist at the same time. Different combinations of the molecular clearance methods are used to achieve different CRRT modes, such as CVVH, CVVHD, CVVHDF and SCUF. There are several methods to determine the amount of fluid being removed. One possible method is to assume that the fluid delivered/removed is equal to the rate of the infusion pumps. This assumption may be inaccurate as the infusion pumps are actually flow restrictors. Sucosky *et al*. reported a standard error in net ultrafiltrate volume removed from the patient of up to 848.5 ± 156 ml over a period of 24 hours in laboratory experiments [30].

Measuring the actual volume of the ultrafiltrate removed by weight or using a volumetric measuring device could be the most precise method. However, this requires strict control by the nursing staff and an inevitable increase in the nursing workload. Another defect is the absence of pressure monitoring in the hemofiltration circuit, which may lead to delayed detection of clotting or rupture of the filter.

##### *Introduction of a CRRT device into the ECMO circuit*

The ECMO circuit can serve as a platform for additional organ support therapies. It is possible to connect a CRRT device to the venous limb of the ECMO circuit before the pump, which drives the blood from the ECMO circuit into the CRRT device. After blood purification the blood is returned to the ECMO circuit before the ECMO pump. If a centrifugal ECMO pump is used, it is necessary to place the CRRT machine after the pump because of the risk of air entrapment. Reconnection to return blood from the CRRT device is required before the oxygenator to trap air or clots before return to the patients, and to avoid venous admixture due to the shunt. Santiago *et al*. reported that ECMO and CRRT devices functioned correctly in all cases after connection of the CRRT device to the ECMO circuit. No pressure changes were observed before or after the inclusion of the CRRT device in the ECMO circuit. Longer filter life was achieved with this method than when CRRT was performed through an independent venous access [27]. Symons *et al*. reported that introduction of a CRRT device into the ECMO circuit provides more accurate fluid management during ECMO [31].

#### Indications for ECMO and ECMO mode selection

Six studies used VA-ECMO alone [13,15,18,22,24,25], predominantly in patients with heart disease. The main indications for ECMO were heart failure and cardiac arrest. Another six studies used both VV-ECMO and VA-ECMO [14,16,19,20,23,26], of which, the main indications were heart failure in three studies [19,20,23], respiratory failure in one study [16] and both heart and respiratory failure in two studies [14,26]. The femoral artery and the femoral vein were the preferred sites for ECMO access in adults, while in pediatric patients, neck vascular access was more likely to be chosen.

#### Indications for CRRT and CRRT mode selection

Nine studies reported the indications for CRRT treatment, including acute renal failure or AKI [16,17,22,24,27], FO [14-17,19,27,29] and metabolic disturbance such as hyperkalemia, electrolyte disturbances or azotemia [14-16,29]. CVVH was employed in five studies [16-19,26], while two studies employed CVVHD and two studies used CVVHDF [13,23]. Multiple modes, including CVVH, CVVHD, CVVHDF and SCUF, were selected in three studies [14,27,28]. Eleven studies used the in-line hemofilter method to provide CRRT [13-20,22,23,26], two of which reported using CRRT devices in cases where ultrafiltration exceeded two liters per hour [17,19]. Three studies employed a CRRT device connected to the ECMO circuit [27,28,31].

#### In-hospital mortality

**Figure 4:**
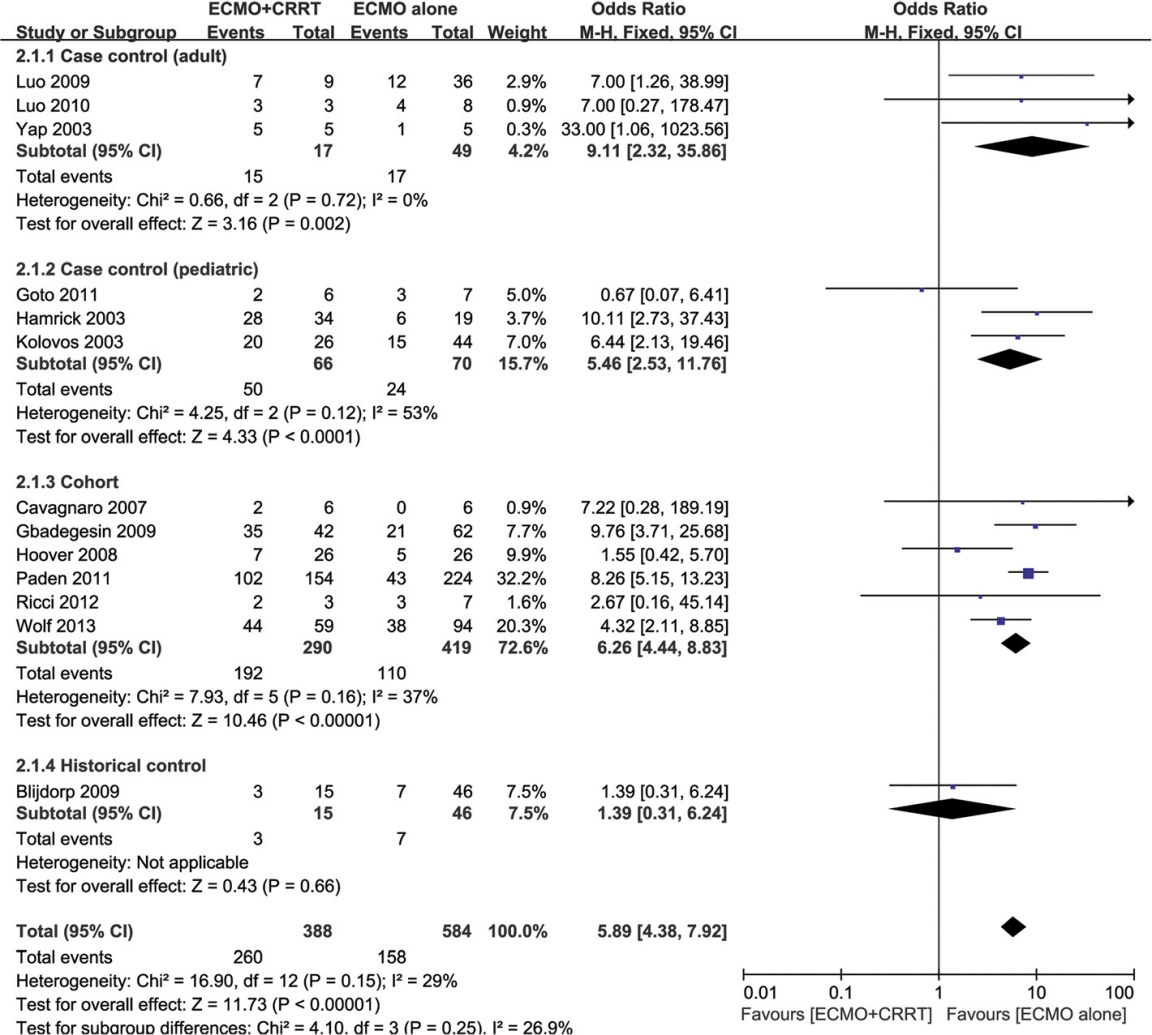
**Forest plot of in-hospital mortality.**

**Figure 5:**
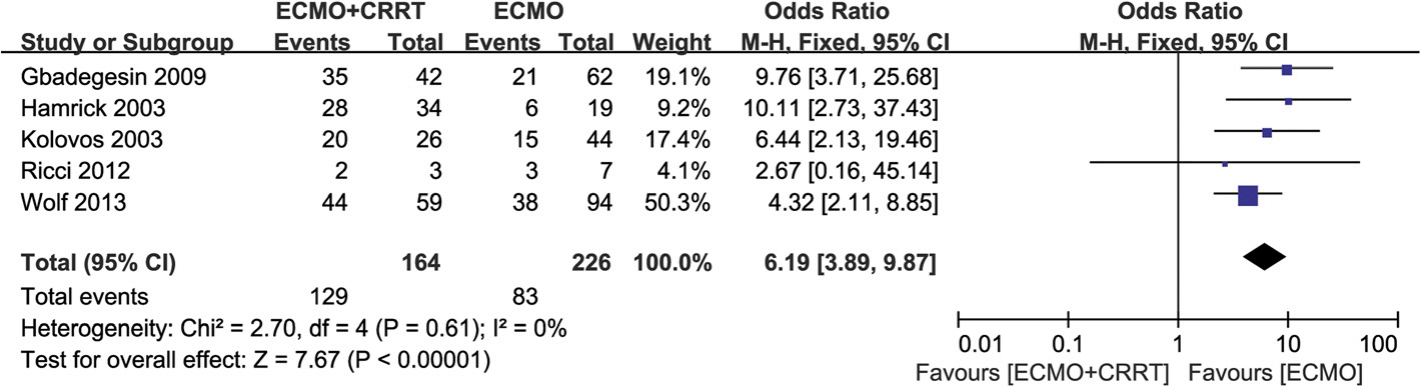
**Forest plot of in-hospital mortality in children with congenital heart disease.**

Thirteen studies reported in-hospital mortality [14-26], with a statistically significant increase in the risk of mortality in ECMO + CRRT patients compared with patients receiving ECMO only (OR 5.89, 95%CI 4.38 to 7.92, *P* <0.00001, Figure 4). There was evidence of mild heterogeneity across the studies (*I*^2^ = 29%) although additional sensitivity analysis showed no heterogeneity (*I*^2^ = 0) after two studies were excluded [16,26]. In one study, patients were matched for age, weight, diagnosis and ECMO mode [26]; in the other study, patients were matched for similar age (±1 year) and similar PRISM III scores at the time of ECMO cannulation (±3) [16]. The OR was 6.82 (95%CI 4.97 to 9.36, *P* <0.00001), after the two studies were excluded (data not shown). We intended to analyze the relationship between different ECMO modes (VA or VV) and mortality, but unfortunately, we were unable to acquire the required data despite our attempts to contact the authors. Figure 5 shows the mortality rate in children with congenital heart disease, with increased risk of mortality observed in ECMO + CRRT patients (OR 6.19, 95%CI 3.89 to 9.87, *P* <0.00001).

#### Fluid balance

Four studies compared fluid balance between ECMO and ECMO + CRRT groups [14,16,22,26]. These showed that for ECMO survivors receiving CRRT, overall fluid balance was less than that in non-CRRT survivors. Gbadegesin *et al*. reported less fluid balance in survivors than in non-survivors who received ECMO therapy [15]. Symons *et al*. found that the in-line CRRT device allowed more accurate fluid management during extracorporeal life support, which may contribute to the shorter duration of extracorporeal life support [31].

#### Renal function recovery

Four studies reported the recovery of renal function before hospital discharge [14,16,17,19]. Three studies showed a full-recovery or no requirement for additional RRT in all survivors before hospital discharge [14,16,19]. Paden *et al*. reported the recovery of renal function and discontinuation of renal replacement in 96% (65/68) of patients before hospital discharge. The follow-up showed that one neonatal patient had normal creatinine one month later. Two pediatric patients developed end-stage renal disease; one received PD and subsequent renal transplant, while the other had diminished function without a requirement for RRT [17].

#### ECMO duration

Eight studies compared the ECMO duration between ECMO and ECMO + CRRT groups [13-19,26]. Blijdorp *et al*. reported that the ECMO duration was shorter in the ECMO + CRRT group [26], whereas another seven studies showed a longer ECMO duration when CRRT was applied.

#### Complications

Bleeding was reported in three studies [20,23,24], including intracranial hemorrhage and gastrointestinal bleeding. Plasma free hemoglobin (FHb) was measured in two studies [13,15]. The ECMO + CRRT group had a higher peak FHb level compared with the ECMO alone group, which indicated that there is enhanced hemolysis during combined ECMO and CRRT compared with ECMO alone. Infection was reported in three studies [23-25]. Cavagnaro *et al*. reported acute pre-renal insufficiency associated with excessive ultrafiltration, which was corrected quickly by decreasing the ultrafiltration rate and stopping the diuretics.

## Discussion

There is an important cardiorenal interaction in patients with either acute or chronic severe heart failure, with renal function commonly decreased in such patients. There is also evidence that both acute and chronic renal dysfunction might contribute to myocardial damage [32]. ECMO is a lifesaving technique used in critical care patients presenting acute cardiac and/or pulmonary dysfunctions. It has been reported that FO during ECMO support is associated with increased risk of mortality [33]. CRRT is an important and efficient therapy that can be combined with ECMO to treat AKI and FO.

There is wide variation in the application of CRRT during ECMO. Besides the three methods we have described previously, CRRT can also be performed under gravity. A ‘Y’ tube system is used to deliver dialysis fluid, with two plastic locks used to control the dialysis flow speed. Effluent fluid is collected in two five-liter bags located below the patient. Ponte *et al*. reported the application of this method in a 22-year-old man, and achieved a zero net balance throughout the procedure [34]; in fact, this method could be considered as a variant of the in-line hemofilter method. Yap *et al*. reported a different direction of blood flow through the hemofilter in their study, in which the inlet catheter of the hemofilter was connected to the proximal limb (before the pump) of the ECMO circuit and the outlet, to the distal limb (after the pump) [22]. The results of an international cross-sectional survey show that 21.5% (14/65) of centers used an in-line hemodiafilter exclusively and 50.8% (33/65) of centers exclusively introduced a CRRT device into the ECMO circuit [8]. The most frequently reported indications were FO (43%), prevention of FO (16%), AKI (35%), electrolyte disturbances (4%) and ‘other’ (2%).

A higher mortality rate was observed after data synthesis, which indicates that the requirement for renal placement therapy could be the risk factor for mortality. However, although mild heterogeneity was observed in our study, additional sensitivity analysis showed no heterogeneity after the exclusion of two studies that had matched the baseline data of patients. No difference in survival was observed in these two studies, which suggests that adding CRRT to ECMO in a non-randomized fashion reflects a relatively higher severity of illness in patients. Data from an entirely heterogeneous group of patients in terms of age and diagnosis may lead to confounding effects; therefore, a subgroup of children with congenital heart disease was analyzed and similar results were obtained. Thus, it can be speculated that, as a component of multiple organ dysfunction syndrome (MODS), the presence of AKI itself rather than the requirement for CRRT, is the independent risk factor for mortality in critically ill patients undergoing ECMO. The difference in ECMO duration can also be explained in this way.

A recently published paper by Luo *et al*. [35] showed that AKI is associated with in hospital mortality in critically ill patients, no matter if it was defined by RIFLE, AKIN or KDIGO criteria. Worse outcome was associated with increased severity of AKI. In populations needing ECMO, a similar situation was observed [36,37]. In a cohort study by Zwiers *et al*. [37] using RIFLE criteria, two thirds of neonates receiving ECMO had AKI, and the mortality risk in the Failure category was significantly increased. We attempted to assess differences in AKI between ECMO and ECMO plus CRRT groups; however, few data were available and, thus, we were unable to finish the assessment.

In both adults and children, a number of studies have shown that a lower FO index at CRRT initiation improves survival [33,38-40]. It has been reported that cumulative FO is independently associated with mortality, worse oxygenation, prolonged ICU stay and duration of mechanical ventilation in critically ill patients receiving CRRT [9]. CRRT provides an easily initiated and efficient method of fluid management. The potential benefit of CRRT and improved fluid balance is indicated by some theoretical advantages, such as enhancing the removal of inflammatory mediators; allowing for more aggressive intervention with nutritional support and achieving more rapid optimal caloric intake [23].

Hemolysis could be a specific complication of combining ECMO and CRRT, with erythrocyte fragmentation caused by the combination of shear stress, positive pressure, wall impact forces and properties of non-endothelialized surfaces [41]. The ECMO + CRRT group had a higher peak FHb level compared with the ECMO alone group in the two studies in which hemolysis was investigated. A higher peak FHb level could be a negative predictor of the change in renal function, possibly due to adverse effects of excess FHb on multi-organ system function. The tetrameric hemoglobin dissociates into dimers after release from cells, which are easily filtered by the glomeruli [42]. FHb precipitates and causes intra-tubular obstruction under acidic conditions, especially with volume depletion, resulting in acute tubular necrosis and acute renal failure. Furthermore, Gbadegesin *et al*. reported that the circulating FHb adversely affects cardiac recovery and the removal from ECMO [15]. A retrospective study of children receiving ECMO showed that, after controlling for baseline data, a higher mortality rate and longer ECMO duration was observed in children presenting hemolysis [43].

Despite the presence of hemolysis, the recovery of renal function seems to be satisfactory. In the absence of primary renal disease presentation, chronic renal failure did not occur in ECMO patients treated concomitantly with CRRT. In the four studies that reported the recovery of renal function, only one reported a case of a 15-year-old male who developed pulmonary hemorrhage, AKI and respiratory failure. The renal biopsy demonstrated microscopic polyangiitis and the patient received a renal transplant due to irreversible renal injury [17]. This finding is also suggested by Meyer *et al*. [44]. In their study, most of the survivors (93%) showed recovery of renal function without a continuing need for RRT. The only exception was a patient with primary renal disease, in whom poor renal function was probably unrelated to either ECMO or CRRT. This finding could encourage less reticence in the utilization of CRRT during ECMO.

This study has important limitations. There could be greater heterogeneity in a systematic review of non-randomized studies than that of randomized trials [12]. Furthermore, it is recommended that the results from different study designs should be expected to differ systematically; hence, we conducted a review with limited data synthesis rather than a formal meta-analysis, and divided enrolled studies into subgroups according to the study designs or according to the age and diagnosis. Nevertheless, the trend in the mortality rate was similar in the different subgroups. It should be emphasized that, due to the heterogeneity, the pooled data should be considered as a clinical reference/hint rather than a validated conclusion. Further prospective studies are needed to determine whether this technique can improve the outcome of critically ill patients. It has been demonstrated that combining ECMO and CRRT may enhance hemolysis [13,15]; however, there were no studies comparing the difference in the occurrence of hemolysis between the methods using an in-line hemofilter and those that combined a CRRT device.

## Conclusions

The combination of ECMO and CRRT might be a safe and effective technique that improves fluid balance and ameliorates electrolyte disturbances. A variety of methods for combining ECMO and CRRT can be chosen. A prospective multicenter study would be beneficial in determining the potential of this technique to improve the outcome of critically ill patients.

## Key messages

- Nineteen studies were included in this systematic review to assess the feasibility, efficacy and safety of the combination of ECMO and CRRT and to illustrate the indications and methodology of providing renal replacement therapy during the ECMO procedure.
- There are three major methods for performing CRRT during ECMO: ‘independent CRRT access’, ‘introduction of a hemofiltration filter into the ECMO circuit (in-line hemofilter)’ and ‘introduction of a CRRT device into the ECMO circuit’.
- The combination of ECMO and CRRT might be a safe and effective technique that improves fluid balance and ameliorates electrolyte disturbances.
- Prospective studies are warranted to determine the potential of this technique to improve the outcome in critically ill patients.

## Additional file

Additional file 1:
**Technical parameters of included studies.**

## Abbreviations

AKI: acute kidney injury
CAVH: continuous arteriovenous hemofiltration
CAVHD: continuous arteriovenous hemodialysis
CAVHDF: continuous arteriovenous hemodiafiltration
CI: confidence interval
CINAHL: Cumulative Index of Nursing and Allied Health Literature
CRRT: continuous renal replacement therapy
CVVH: continuous venovenous hemofiltration
CVVHD: continuous venovenous hemodialysis
CVVHDF: continuous venovenous hemodiafiltration
ECMO: extracorporeal membrane oxygenation
FHb: free hemoglobin
FO: fluid overload
ICU: intensive care unit
MODS: multiple organ dysfunction syndrome
NOS: Newcastle - Ottawa quality assessment scale
OR: odds risk
PD: peritoneal dialysis
RCT: randomized controlled trial
RRT: renal replacement therapy
SCUF: slow continuous ultrafiltration
VA-ECMO: venoarterial extracorporeal membrane oxygenation
VV-ECMO: venovenous extracorporeal membrane oxygenation
