# Supplementary material for: Combination of extracorporeal membrane oxygenation and continuous renal replacement therapy in critically ill patients: a systematic review
Source: Crit Care. 2014 Dec 8;18(6):675. doi: 10.1186/s13054-014-0675-x (PMC4277651; doi:10.1186/s13054-014-0675-x)
Supplement: Additional file 1: — Technical parameters of included studies. [file 13054_2014_675_MOESM1_ESM.docx]

**Additional file 1 Technical parameters of included studies**

| Author; year | Indications for ECMO | ECMO access | VA/VV-ECMO | ECMO pump | ECMO flow rate | Anticoagulation | CRRT indications | Connection method | CRRT mode |
| --- | --- | --- | --- | --- | --- | --- | --- | --- | --- |
| Goto;  2011 | Circulatory collapse;  profound hypoxemia | Right atrium and ascending aorta | 10VA 2VV 2VA→VV | Roller pump;  Centrifugal pump | 0-120 ml/kg/min | Heparin ACT 150-250s | NR | In-line hemofilter | CVVHDF |
| Hamrick;  2003 | Failure to wean from bypass; cardiac arrest; arrhythmia; slow deterioration | NR | NR | NR | NR | NR | NR | NR | CAVH |
| Kolovos;  2003 | Ventricular failure; Pulmonary failure; Increased PVR; Multiple indications; Shunt occlusion; | Neck; groin; chest | 73VA 1VV | NR | NR | Heparin ACT 180-200s | NR | In-line hemofilter | CVVHD |
| Luo;  2009 | Heart disease after surgery; chronic heart failure | Femoral vein and femoral femoral artery; axillary artery and femoral vein;  right atrium and ascending aorta; | VA | NR | 40ml/kg/min | Heparin ACT 150s | ARF | NR | NR |
| Luo;  2010 | Cardiac arrest | Femoral vein and femoral femoral artery | VA | NR | 40ml/kg/min | Heparin ACT 150s | NR | NR | NR |
| Yap;  2003 | Heart Failure | Femoral vein and femoral femoral artery | VA | Centrifugal pump | 4-4.5L/min | Heparin ACT 160-180s | ARF | In-line hemofilter* | CVVHDF |
| Betrus;  2007 | Systolic and/or diastolic ventricular dysfunction; respiratory failure; occluded systemic-to-pulmonary artery shunt. | Internal jugular vein and carotid artery;  right atrium and ascending aorta | VA | Roller pump | 80-120 ml/kg/min | Heparin ACT 180-200s | NR | In-line hemofilter | CVVHD |
| Cavagnaro;  2007 | Cardiac and/or respiratory failure | Jugular vein;  internal jugular vein and carotid artery | 6VA 6VV(1→VA) | Roller pump | 150 ml/kg/min | Heparin ACT 200-200s | FO azotemia | In-line hemofilter | CVVH CVVHDF SCUF |
| Gbadegesin;  2009 | Systolic and/or diastolic ventricular dysfunction; respiratory failure; occluded systemic-to-pulmonary artery shunt. | Internal jugular vein and carotid artery | VA | Roller pump | 80-120 ml/min/kg | Heparin ACT 180-200s | FO hyperkalemia | In-line hemofilter | NR |
| Hoover;  2008 | Respiratory failure | NR | 6VA 46VV | NR | NR | NR | FO Electrolyte imbalance ARF | In-line hemofilter | CVVH |
| Paden;  2011 | Cardiac and/or respiratory failure; ECMO cardiopulmonary resuscitation | NR | NR | NR | NR | NR | FO AKI | In-line hemofilter;  CRRT device** | CVVH |
| Ricci;  2012 | Cardiac dysfunction; respiratory failure | Femoral vein and femoral femoral artery;  jugular vein and carotid artery;  transthoracic | VA | NR | NR | NR | NR | In-line hemofilter | CVVH |
| Wolf;  2013 | NR | NR | 149VA 4VV | Roller pump | NR | NR | FO | In-line hemofilter;  CRRT device** | CVVH |
| Blijdorp;  2009 | Congenital diaphragmatic hernia; meconium aspiration syndrome | NR | 57VA 4VV | Roller pump | 120-150 ml/min/kg | NR | NR | In-line hemofilter | CVVH |
| ECMO=extracorporeal membrane oxygenation; VV=venovenous ECMO; VA=venoarterial ECMO; ACT=active clotting time; PVR=pulmonary vascular resistance; FO=fluid overload; ARF=acute renal failure; AKI=acute kidney injury; NR=not reported; CVVH=continuous venovenous hemofiltration; CVVHD=continuous venovenous hemodialysis; CVVHDF=continuous venovenous hemodiafiltration; CAVH=continuous arteriovenous hemodialysis; SCUF=slow continuous ultrafiltration; URF=ultrafiltration rate * Different blood flow direction ** If UFR exceeded 2 L/h | | | | | | | | |  |
